# Androgen Receptor in Breast Cancer: From Bench to Bedside

**DOI:** 10.3389/fendo.2020.00573

**Published:** 2020-09-02

**Authors:** Mengyao Chen, Yunben Yang, Kai Xu, Lili Li, Jian Huang, Fuming Qiu

**Affiliations:** ^1^Department of Medical Oncology, The Second Affiliated Hospital, Zhejiang University School of Medicine, Hangzhou, China; ^2^Key Laboratory of Tumor Microenvironment and Immune Therapy of Zhejiang Province, The Second Affiliated Hospital, Zhejiang University School of Medicine, Hangzhou, China; ^3^Cancer Institute, Key Laboratory of Cancer Prevention and Intervention, Ministry of Education, The Second Affiliated Hospital, Zhejiang University School of Medicine, Hangzhou, China; ^4^Department of Orthopedics Surgery, The Second Affiliated Hospital, Zhejiang University School of Medicine, Hangzhou, China; ^5^Department of Breast Surgery and Oncology, The Second Affiliated Hospital, Zhejiang University School of Medicine, Hangzhou, China

**Keywords:** breast cancer, androgen receptor, prognosis, targeted therapy, drug resistance

## Abstract

Breast cancer (BC) is one of the most common malignancies and the leading cause of cancer-related mortality in women. Androgen receptor (AR) is frequently expressed in diverse BC subtypes. Accumulating evidence has revealed that AR might be a predictive or prognostic factor and a drug target in BC. AR expression and AR pathways differ in various BC subtypes, thereby resulting in controversial inferences on the predictive and prognostic value of AR. Herein, we summarized the roles of AR in different BC subtypes and AR-targeting therapies based on preclinical and clinical studies. Moreover, we highlighted the possible efficacy of a combination therapy via exploiting the AR-related mechanisms and the research on therapeutic resistance.

## Introduction

Breast cancer (BC) represents the most common malignancy in women, and is one of the leading causes of cancer-related fatality (11.6% of the total cancer deaths) (1). BC has different subtypes that are usually classified depending on the expression of the estrogen receptor (ER), progesterone receptor (PR), and human epidermal growth factor receptor 2 (HER2). ER-positive (ER+) BC is the most common subtype, comprising 70% of all BC cases (2, 3), while HER2-positive (HER2+) BC accounts for 20–25% (4). Although such patients can benefit from treatment targeting the ER or HER2, ER+ BC frequently acquires resistance to endocrine therapy (5). Triple-negative BC (TNBC), which lacks the expression of ER, PR, and HER2, constitutes 15–20% of BC cases, and has the highest probability of metastasis and the lowest overall survival (OS) among all BC subtypes (6, 7). Currently, there is no well-defined targeted therapy for TNBC (8). Given the urgency in the development of effective BC treatment strategies, it is essential to identify new markers or potential alternative therapeutic targets for this disease in order to predict prognosis, diminish drug resistance, and improve clinical outcomes.

Androgen receptor (AR), a member of the steroid receptor superfamily, is expressed in many human tissues, among which BC tissue has the third-highest expression of AR (8, 9). It has been noted that 70–90% of BC patients overexpress the AR with several studies indicating that AR might be a predictive or prognostic factor and a drug target in BC (10). Recently, the implication of AR in various stages of BC gathered significant attention. Although there have been several reviews discussing the physiology of AR, AR-related mechanisms, and AR-targeting treatments, its function appears to vary among the diverse BC subtypes, and its prognostic and predictive value in BC patients remains controversial (11, 12).

In this review, we focused on the roles and prognostic significance of AR and AR-targeting therapies in different BC subtypes based on preclinical and clinical studies. We also summarized the possibility of combination treatment and research on therapeutic resistance.

## Methods

We performed a comprehensive search of the PubMed database for articles written in English and published before January 2020 (mainly after 2010), using the following search terms, alone or in combination: “androgen receptor,” “androgen,” “breast cancer,” “prognosis,” “androgen receptor agonist or antagonist,” and “resistance.” In addition, bibliographies of relevant articles were searched for additional references. The website ClinicalTrials.gov was searched for ongoing clinical trials.

## Roles of AR in BC

AR is a 919-amino-acid hormone-regulated transcription factor. The gene responsible for AR measures 180 kb in length and is located on the chromosome, Xq11-12 (13). AR comprises four distinct functional domains: (i) an N-terminal domain (NTD), in charge of transcriptional activity; (ii) a DNA-binding domain (DBD) that primarily recognizes specific androgen response elements (AREs); (iii) a hinge region for nuclear localization, and (iv) a ligand-binding domain (LBD) for androgen binding (14). Androgens are steroid hormones that regulate AR transcriptional activity via AR binding (15). However, among the androgens, only testosterone (T) and dihydrotestosterone (DHT) can bind the AR (15, 16). In the breast tissue, T is converted either to DHT by 5α-reductase or to 17β-estradiol (E2) by aromatase (17). Interestingly, DHT concentrations were significantly (3-fold) higher, whereas 5α-reductase levels were elevated 4- to 8-fold in breast carcinoma tissues compared with those in non-neoplastic breast tissues (16). Of note, T is converted into estradiol only when the estrogen levels are very low (15, 17).

**Figure 1:**
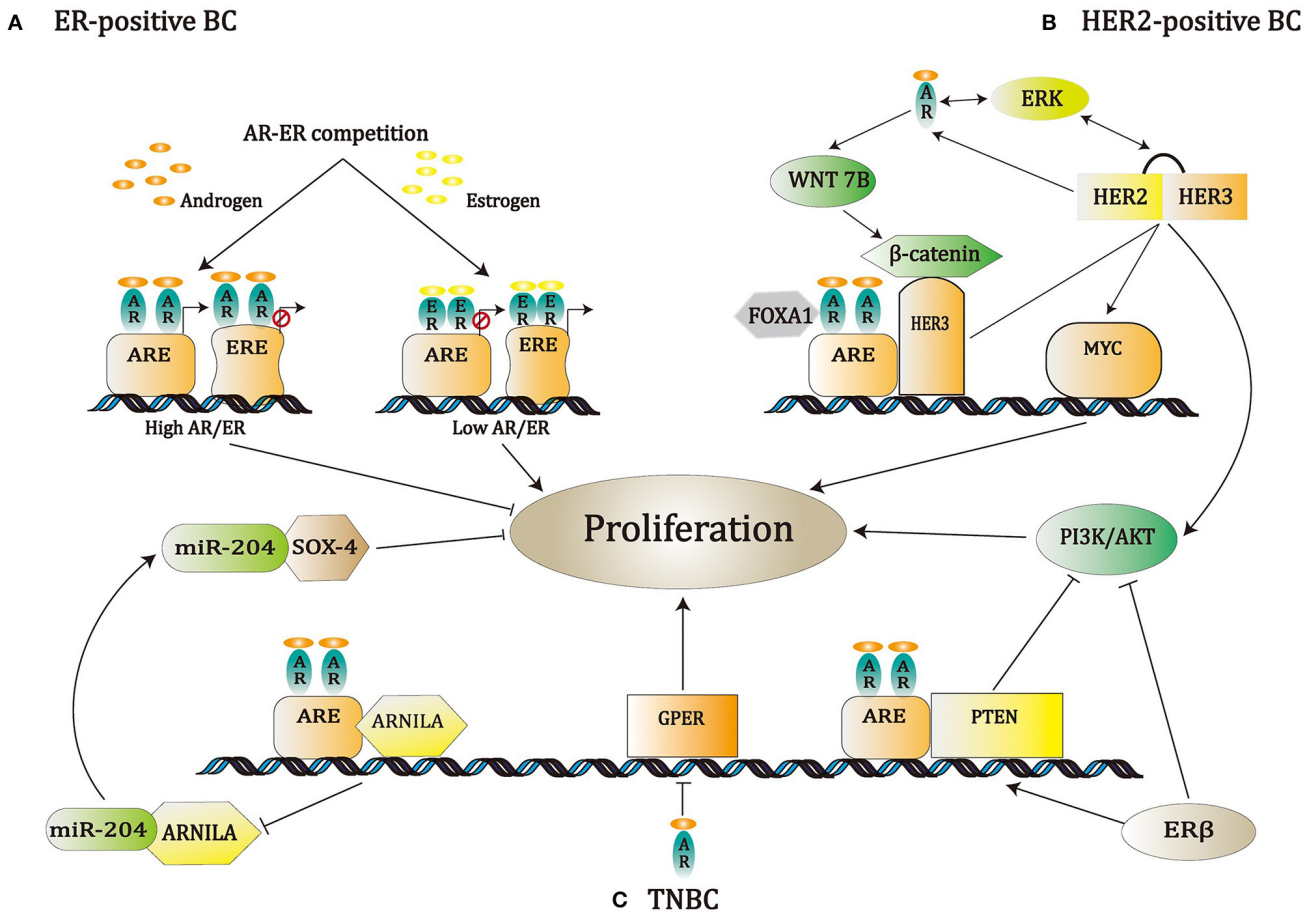
A schematic of known pathways regulated by AR in various breast cancer subtypes. **(A)** In ER-positive BC, it has been found that AR influenced the ER pathway by competition with ER for attaching to the modulatory regions of ER genes; meanwhile, the AR/ER expression ratio makes a critical difference in the regulation of BC cell proliferation. **(B)** In HER2-positive BC, the Wnt/β-catenin signaling pathway is up-regulated by the AR. AR/β-catenin complex, together with FOXA1, induces HER3 gene transcription. Next, HER3 and HER2 create a heterodimer, thereby stimulating the PI3K/AKT pathway and MYC gene expression. Additionally, ERK, AR, and HER2 take part in a positive feedback cycle. **(C)** In TNBC, the up-regulation of PTEN through AR or ERβ represses PI3K action, which in turn reduces AR activity. Suppression of GPER by AR can also modulate cell behavior positively, while suppression of ARNILA results in negative modulation.

AR is situated in the cytoplasm, and without the ligand, it adheres to heat shock proteins (HSPs) (14). Once androgens enter the cell, they combine with the AR resulting in conformational changes that enable its dimerization. Subsequently, AR is isolated from HSPs and transferred to the nucleus. In the nucleus, the DBD attaches to AREs within the target gene and recruits additional co-stimulators, co-repressors, and transcriptional regulators that positively or negatively influence gene transcription, thus altering apoptosis, differentiation, angiogenesis, or proliferation (18, 19). AR activity can be modulated by several key proteins and signaling pathways, such as Wnt/β-catenin, PI3K/AKT pathways, phosphatase and tensin homolog deleted on chromosome 10 (PTEN), forkhead box protein A1 (FOXA1), G-protein coupled estrogen receptor (GPER), as well as long non-coding RNA (lncRNA). Moreover, the action of AR is also associated with ER and HER2 signaling pathways (20–25). The crosstalk between AR and other pathways or proteins is described in detail in the following sections and depicted in Figure 1.

### AR in ER-Positive BC

In ER-positive BC, AR has been demonstrated to interfere with ER-dependent transcription through competition with the ER for binding sites at the estrogen response elements (EREs) or through competition for the transcriptional co-modulators (23). Indeed, the AR/ER expression rate determines the impact of both AR and ER in the regulation of BC cell proliferation. When the expression level of AR is higher than that of ER, the AR binds to the EREs, thereby exerting inhibitory effects on cell growth. In contrast, if the ER is more abundant than the AR, then ER binds to AREs, leading to cell proliferation (Figure 1) (15, 26–28).

### AR in HER2-Positive BC

In HER2-positive BC, AR transcriptionally regulates the levels of WNT7B that leads to the transfer of β-catenin into the nucleus. In the nucleus, the AR/β-catenin complex identifies the modulatory regions of HER3 and raises its transcription under the cooperation of the transcription factor, FOXA1. HER3 and HER2 form a heterodimer that stimulates MYC gene expression and PI3K/AKT pathway, thereby resulting in cell proliferation and tumor growth (Figure 1) (29). The synergism between AR and HER2 is boosted via the mechanism in which HER2 promotes AR transcription and leads to ERK activation that, in turn, regulates both HER2 and AR, resulting in a positive feedback loop (Figure 1) (24).

### AR in TNBC

It is known that the PI3K/AKT/mTOR pathway is involved in BC development (30). In TNBC, the frequency of PIK3CA mutations in AR+ tumors was higher than that in AR-negative tumors (31). Androgen up-regulates PTEN transcriptional expression when AR binds to AREs in the PTEN upstream promoter. Subsequently, PTEN restrains PI3K action, which in turn weakens AR activity. Nevertheless, in prostate cancer, AR binds to the PTEN promoter as a repressor, thereby inhibiting its transcription (32). Elevated PIK3CA protein activity in AR+ TNBC (through amplification and mutation) will bypass the AR-mediated PTEN up-regulation so that targeting AR alone may promote tumor growth by lowering PTEN expression and motivating the PI3K pathway. Therefore, dual targeting of the AR and PI3K may produce a synergistic anti-tumor effect (31).

ERβ, a second form of estrogen receptor, is found in ~30% of TNBC cases (33). ERβ overexpression can decrease AR activation by the up-regulation of PTEN and the suppression of the PI3K/AKT signaling pathway (34, 35). GPER is a member of the G-protein coupled receptor (GPCR) family, which is primarily expressed in TNBC and reportedly regulates the development of various cancer types (36). Notably, the activation of AR may promote the proliferation of TNBC by down-regulating GPER (25).

Additionally, in TNBC, it was found that AR could adjust the cell behavior via modulating the interaction between microRNAs and lncRNAs. The AR negatively induced lncRNA (ARNILA) was reported to serve as a competitive endogenous RNA of miR-204 that promotes the expression of its target gene, Sox4, which boosts epithelial–mesenchymal transition (EMT), invasion, and metastasis of TNBC. Therefore, ARNILA is associated with poor progression in TNBC (22).

## The Clinical Relevance of AR in BC

AR can be detected in most BCs, and the frequency of its positivity differs between different BC subtypes and ethnicities (10, 37–42). Numerous studies exploring the prognostic value of AR in BC have been presented with contentious results (Table 1); in fact, the AR effect depends on tumor subtypes or patient populations.

**Table 1:** Prognostic value of AR in different breast cancer subtypes.

**References**	**AR detection**	**AR antibody**	**Cut-off**	**Patients**	**Endpoint**	**Subtype**	**Result**
Kensler et al. (43)	IHC	AR441(DAKO)	≥1%	4,147	BCS, OS, RFS	ER-positive ER-negative	Improved survival Worse survival
Bozovic-Spasojevic et al. (44)	IHC	NA	NA	10,004	DFS, OS	ER-positive Her2-positive TNBC	Improved DFS and OS Not associated with DFS, but worse OS Improved DFS and OS
Okano et al. (45)	mRNA Z-scores	NA	NA	NA	pCR, DRFS	ER-positive	Worse response to NAC, but better survival
Kensler et al. (46)	IHC	AR441(DAKO)	≥1%	3,021	DFS	ER-positive	Not associated with prognosis
Cochrane et al. (47)	IHC	AR441(DAKO)	≥2.0*	192	DFS	ER-positive	Poor response to endocrine therapy
Wang et al. (48)	IHC	ZA-0554	≥10%	304	PFS, OS	Her2-positive	Prolonged PFS and OS
Kucukzeybek et al. (49)	IHC	AR441(DAKO)	≥7.5%	111	DFS, OS	Her2-positive TNBC	Not associated with prognosis Longer OS
Asano et al. (50)	IHC	AR441(DAKO)	≥1%	190	RFS, CSS	TNBC	Better prognosis
Yang et al. (22)	IHC	Ab1983394	NA	88	PFS	TNBC	Prolonged PFS
Hilborn et al. (51)	IHC	AR441(DAKO)	≥1%	912	RFS	ER-negative Her2-positive	Improved outcome with tamoxifen Could not predict outcome with tamoxifen
Xu et al. (52)	IHC	NA	NA	4,914	DFS,OS, DDFS, RFS	TNBC	Not associated with prognosis
Speers et al. (53)	Data set	NA	NA	283	LRFS	TNBC	Worse LRFS after radiation therapy
Loibl et al. (54)	IHC	F39.4.1Nuc AM256-2ME (RTU-M)	>51%	673	DFS, OS, pCR	ER-positive Her2-positive TNBC	Not associated with prognosis Not associated with prognosis Better DFS and OS, low chance of pCR
Bhattarai et al. (55)	IHC	AR441(DAKO)	≥1%	1,047	OS	TNBC	OS present population-specific patterns
Elebro et al. (56)	IHC	AR441(DAKO)	>75%	905	DFS	ER-positiveER-negative	Concordant AR and ER expression was associated with superior prognosis

ER, estrogen receptor; AR, androgen receptor; HER2, human epidermal growth factor receptor 2; TNBC, triple-negative breast cancer; NA, not available; IHC, immunohistochemistry; BCS, breast cancer-specific survival; RFS, recurrence-free survival; DFS, disease-free survival; OS, overall survival; PFS, progression-free survival; CSS, cancer-specific survival; DDFS, distant disease-free survival; LRFS, local recurrence-free survival; pCR, pathologic complete response. NAC, neoadjuvant chemotherapy; *the AR:ER ratio cut-off point.

### ER-Positive BC

AR is expressed in 70–95% of ER+ BCs (10, 37, 38). AR has been noted to be correlated with favorable outcomes, such as smaller tumor size, lower tumor grade, less necrosis, lower Ki-67 levels, and better treatment response in ER+ BC (39, 57–59). Recently, a large study including 4,147 pre- and post-menopausal women with invasive BC from the Nurses' Health Study cohorts showed that AR expression, independent of clinicopathological characteristics, was related to the improvement of BC-specific survival in ER+ BC patients in the first 5–10 years post-diagnosis (43). A meta-analysis by Bozovic-Spasojevic et al. employing univariate and multivariate analyses, uncovered that AR expression results in significantly improved disease-free survival (DFS) and OS in ER+ BCs (44). In one study using transcriptomic data obtained from TCGA and METABRIC cohorts, high AR levels in ER+ BC correlated with fewer tumor-infiltrating lymphocytes and cytolytic activity, as well as far less sensitivity to neoadjuvant chemotherapy, yet better survival (45). However, a study of 3,021 postmenopausal women with early-stage ER+ BC showed that AR expression was not linked with the prognosis, nor could it predict the response to letrozole or tamoxifen (46).

On the contrary, the AR/ER ratio has been considered to affect the prognosis and response to anti-estrogen endocrine therapy. Cochrane et al. revealed that ER+ BC patients with an AR/ER ratio > 2, had a 4-fold increased risk of failure with tamoxifen therapy (47).

### HER2-Positive BC

Approximately 30–60% of HER2+ BCs overexpress the AR (10, 40). Naturally, HER2+ BC patients show poorer prognosis compared to HER2-negative BC patients (60). However, when HER2, ER, and AR were all expressed, the tumor acted with a weak invasive phenotype, and the patient had a superior outcome (61). A study comprising of 304 HER2-enriched metastatic BC (MBC) cases, considered that AR positivity was an independent prognostic marker for progression-free survival (PFS) (HR = 0.71, *P* = 0.039) and OS (HR = 0.53, *P* = 0.013). Besides, patients treated with first-line trastuzumab, AR+ tumors had longer PFS (15.8 vs. 8.2 months, *P* = 0.005) and 5-year OS rate (66.2 vs. 26.2%, *P* = 0.009) compared with AR-negative subjects (48). In addition, a study involving 111 operated patients with BC revealed no significant correlations between AR expression and prognostic values in the HER2+ group (49). On the contrary, a notable finding of a meta-analysis, including three studies with 358 patients, revealed the worse clinical outcome conferred by AR expression in patients with HER2+ER-(Her2-enriched) BC (44).

### TNBC

In TNBC cases, the expression of AR is 10–53% (39–41); however, the prognostic value of AR continues to be disputable. For instance, an analysis of the immunohistochemical results in 190 TNBC patients demonstrated markedly preferable prognosis (*P* = 0.019) in those with AR+ subtypes than that in those with AR-negative subtypes (50). Another similar analysis of 88 TNBC patients revealed that higher expression of AR was dramatically related to a prolonged PFS (HR = 0.12; *P* = 0.011) (22). Besides, a retrospective analysis showed that the AR status could be used to identify groups of ER-negative BC patients benefiting from adjuvant tamoxifen therapy. In ER-negative BC patients, AR expression predicted reduced recurrence rate with tamoxifen; even in TNBC, patients with AR+ tumors showed an improved outcome when treated with tamoxifen (51).

However, in a meta-analysis of 27 studies, including 4,914 TNBC patients, AR expression was not related to DFS, OS, distant DFS, or recurrence-free survival (52). Moreover, a recent study about peculiar clinical groups, including TNBC patients treated with or without radiation, showed a noticeable correlation between AR expression and locoregional recurrence only in patients who had radiation therapy, suggesting that AR expression might be a marker predicting the response to radiotherapy in TNBC (53). In addition, compared with the primary tumor, AR gene expression increased in circulating tumor cells and early lung metastases, indicating that AR may promote the spread of metastasis by supporting the survival of BC cells during metastasis (62). Several retrospective studies demonstrated that AR+ TNBC patients had an inferior response to chemotherapy and a lower opportunity of achieving a pathological complete response to neoadjuvant chemotherapy (54, 63).

A multi-institutional study of 1,407 TNBC patients from six international cohorts found that AR status presents population-specific patterns related to OS. AR positivity is a biomarker of favorable prognosis in the Nigerian and US cohorts, whereas it correlated with poor prognosis in the Indian, Norway, and Ireland cohorts, while being neutral in the UK cohort (55).

To some extent, the prognostic discrepancy mentioned above may be owing to differences in sample sizes, the methodology of detection, the antibody used to test AR, the cut-off values used to define AR positivity, the ethnic composition of cohorts, adjuvant treatments, and follow-up time of studies (55, 64, 65).

## AR-Related Therapies in BC

### AR-Targeted Monotherapy

Natural and synthetic androgens have been used as a treatment approach in BC with AR expression (12, 66, 67); however, they have been known to induce many side effects (68). The new selective-AR modulators (SARM), as AR agonists, can solve this problem (69). Moreover, AR antagonists have also been investigated extensively in previous studies. The first-generation non-steroidal AR antagonist, bicalutamide, blocks DBD conjugating with the AREs (70, 71). Moreover, bicalutamide possesses partial agonist effects (72). Patients resistant to bicalutamide usually can respond to enzalutamide, a second-generation AR antagonist, which has better anti-tumor efficacy than bicalutamide, because of its higher affinity for AR, ability to inhibit nuclear translocation, gene binding, and recruitment of coregulators (73, 74). Unfortunately, there have been reports of adverse events related to the central nervous system and resistance to enzalutamide (75). Darolutamide (ODM-201) is a novel AR antagonist, which enables the interruption of the tested mutant AR activity in anti-androgen treatments, especially a mutation of F876L in the LBD that is recognized as resistant to enzalutamide; it does not increase serum testosterone levels and exhibits negligible brain penetrance (76). Other novel AR antagonists are under development. For example, orteronel (TAK-700), VT-464 (Viamet), and abiraterone acetate, all CYP17A inhibitors that interrupt the synthesis of androgens through suppressing 17,20-lyase or 17α-hydroxylase activity, are currently under investigation for their therapeutic efficacy in BC (56, 77).

#### ER-Positive BC

So far, androgens and AR agonists have been explored in ER+ BC. A retrospective analysis of ER+ MBC patients revealed that the clinical benefit rate (CBR) of fluoxymesterone was 43% after progressing to conventional hormonal therapy, independent of the AR expression level (67). Enobosarm (GTx-024), one of the new SARM, has shown promising results in the case of an extensively pretreated woman with metastatic ER+/PR+/HER2–BC with high AR co-expression who reached partial radiographic response after four cycles of treatment with enobosarm (69). Currently, a phase II clinical trial investigating enobosarm for locally advanced or metastatic ER+ and AR+ BC patients is under way (NCT02463032) (Table 2).

**Table 2:** AR-targeted clinical trials in breast cancer.

**Identifier**	**Study design**	**Agent activity**	**Agents**	**Study population**	**Patients**	**Endpoint**	**Status**
NCT02463032	Randomized, open label Phase II	Selective-AR modulator	GTx-024	ER+ AR+ BC	88	CBR	Active, not recruiting
NCT03055312	Randomized, open label Phase III	AR inhibitor	Bicalutamide	AR+ metastatic TNBC	262	CBR	Recruiting
NCT01889238	Nonrandomized, open label Phase II	AR inhibitor	Enzalutamide	AR+ advanced TNBC	118	CBR	Active, not recruiting
NCT02750358	Nonrandomized, open label Phase II	AR inhibitor	Enzalutamide	AR+ TNBC Stage I–III	50	1-year dose compliance rate	Active, not recruiting
NCT03383679	Randomized, open label Phase II	AR inhibitor	Darolutamide	AR+ locally recurrent or metastatic TNBC	90	CBR	Recruiting
NCT01842321	Nonrandomized, open label Phase II	CYP17 inhibitor	Abiraterone acetate	AR+ metastatic or locally advanced TNBC	31	CBR	Active, not recruiting
NCT02457910	Randomized, open label Phase Ib/II	PI3K inhibitor/ AR inhibitor	Taselisib/ enzalutamide	AR+ metastatic TNBC	73	CBR	Active, not recruiting
NCT02605486	Nonrandomized, open label Phase I/II	AR inhibitor/ CDK4/CDK6 inhibitor	Bicalutamide/ palbociclib	AR+ metastatic TNBC	51	PFS	Recruiting
NCT02971761	Open label Phase II	Selective-AR modulator/ Anti-PD-1	GTx-024/ pembrolizumab	AR+ metastatic TNBC	29	CR or PR	Active, not recruiting
NCT02689427	Nonrandomized, open label Phase IIB	AR inhibitor/chemotherapy	Enzalutamide/ paclitaxel	AR+ TNBC Stage I-III	37	pCR RCB-I	Recruiting
NCT02091960	Open label Phase II	AR inhibitor/ HER2 Inhibitor	Enzalutamide/ transtuzumab	HER2+ AR+ metastatic or locally advanced BC	103	CBR	Active, not recruiting

*BC, breast cancer; ER, estrogen receptor; AR, androgen receptor; HER2, human epidermal growth factor receptor 2; ER+, ER-positive; AR+, AR-positive; HER2+, HER2-positive; TNBC, triple-negative breast cancer; PI3K, phosphoinositide 3-kinase; CBR, clinical benefit rate; PFS, progression-free survival; pCR, pathologic complete response; CR or PR, complete response or partial response; RCB-I, residual cancer burden-index*.

#### ER-Negative BC

The AR-directed antagonists are mostly used in advanced BC, especially TNBC (12). Preclinical studies detected bicalutamide to inhibit proliferation and increase apoptosis in AR+/ER-negative BC cell lines, and even in the MSL TNBC BC cells, Hs578T and MDA-MB-231, which express relatively low levels of AR (9, 78). A phase II clinical trial comprising 424 ER/PR-negative BC patients, showed the CBR at 6 months of bicalutamide treatment was 19% and a median PFS duration of 12 weeks but no objective responses (79). This trial demonstrated, for the first time, the efficacy of AR-targeted therapy with advanced AR+ TNBC. Currently, there is an ongoing phase III clinical study comparing the effectiveness of bicalutamide with conventional chemotherapy as the first-line treatment of AR+ metastatic TNBC (NCT03055312) (Table 2).

Furthermore, a large preclinical study showed that enzalutamide significantly reduced proliferation, migration, and invasion, while increasing apoptosis in 11 BC cell lines (three non-TN and eight TNBC); moreover, growth inhibition was dependent on the expression of AR, which is consistent with the findings of another research that involved four different non-LAR TNBC cell lines of the MSL, BL2, and mesenchymal-like subtypes demonstrating enzalutamide-mediated cell inhibition (80, 81). A phase II clinical trial of locally advanced or metastatic AR+ TNBC patients showed that the CBR of enzalutamide was 33% at 16 weeks and 28% at 24 weeks, median PFS duration was 3.3 months, and the median OS was 17.6 months in the evaluable subgroup (NCT01889238) (Table 2) (82). Based on these encouraging results, enzalutamide is currently undergoing evaluation for its efficacy in early AR+ TNBC in a phase II clinical trial (NCT02750358) (Table 2).

Beyond that, a randomized phase II study in locally recurrent (unresectable) or metastatic AR+ TNBC treated with darolutamide or capecitabine is currently recruiting patients (NCT03383679) (Table 2) (83). Another phase II trial presented encouraging results in patients with molecular apocrine-like tumors treated with abiraterone acetate plus prednisone (NCT01842321) (Table 2) (84), and the clinical trial is still ongoing.

### Combination of AR-Targeted Therapy With Other Therapies

Aside from monotherapy approaches, the combination of AR-targeted therapy with other therapies as new potential strategies has gathered considerable interest. Previous studies have revealed that in AR+ TNBC, PIK3CA kinase mutations were more common and that targeting AR alone could potentially enhance tumor growth (31). A study demonstrated that the combination of bicalutamide with GDC-0941 or GDC-0980 (pan-PI3K or dual PI3K/mTOR inhibitor) had additive effects in cell line models and xenograft tumors (31). When enzalutamide plus the mTOR inhibitor, everolimus or anti-HER2 monoclonal antibody, trastuzumab were used to treat TNBC and HER2+ cell lines, it resulted in synergistic inhibition of proliferation (85). Lehmann et al. found that the combination of enzalutamide with taselisib, a PI3K inhibitor, significantly increased the CBR of advanced TNBC patients, and was ~35.7%, compared to those treated with enzalutamide alone in a phase IB/II clinical trial (NCT02457910) (Table 2) (86). Interestingly, one patient achieved a partial response to the combination after progressing to enzalutamide alone (86), indicating that combinatorial strategies may be needed for AR+ TNBC patients to obtain a significant clinical benefit in the future.

Cyclin-dependent kinases (CDKs) are a family of protein kinases that play a crucial role in eukaryotic cell proliferation. CDK4 and CDK6, members of the CDK family, are activated by cyclin D and induce the transition from G1 phase to S phase in cell proliferation (87). Palbociclib, a highly selective CDK4/6 inhibitor, combined with enzalutamide cemented the cellular inhibition of AR+/Rb-proficient (MDA-MB-453) TNBC cells, which provided a preclinical rationale for the selection of patients who may benefit from the combined treatment of CDK4/6 inhibitors and AR antagonists (88). At present, a phase I/II trial (NCT02605486) (Table 2) is ongoing to explore the therapeutic effect of combining palbociclib and bicalutamide in metastatic AR+ TNBC.

In the studies involving BC, little research has been done on the association between AR and immune response. In a previous study on prostate cancer, increased PD-L1/2-positive dendritic cells and PD-1-positive T cells were found in the blood of patients progressing to enzalutamide, compared to those naïve or responding to enzalutamide, suggesting that the combination of enzalutamide and anti-programmed death-1 therapy may improve the prognosis in patients with enzalutamide resistance (89). Meanwhile, there is an ongoing clinical trial (NCT02971761) (Table 2) integrating AR-targeted therapy with immunotherapy. In the trial, GTx-024 (SARM) and pembrolizumab are used to treat patients with AR+ metastatic TNBC.

Furthermore, AR antagonists have been combined with conventional therapies, such as radiotherapy and chemotherapy, with agreeable results. For example, Barton et al. in a preclinical model, reported that the combination of enzalutamide with paclitaxel (simultaneous and subsequent) was more effective than paclitaxel alone in reducing tumor recurrence (90). There are several ongoing clinical trials combining chemotherapy and anti-androgen therapy, which are included as neoadjuvant therapy (NCT02689427) (Table 2). As mentioned above, AR positivity was most dramatically correlated with worse local recurrence-free survival after radiotherapy, while enzalutamide significantly radiosensitized AR+ TNBC cell lines and xenograft tumors (53). Besides, there is an ongoing phase II clinical trial (NCT02091960) (Table 2) evaluating the efficacy of enzalutamide combined with trastuzumab in HER2/AR-positive locally advanced or metastatic BC. Several clinical trials currently exploring AR-targeted therapy as mono- or multi-therapy are listed in Table 2.

## Mechanisms of Resistance to AR Antagonists

While the results of AR-targeted therapies for BC are promising, some patients eventually show disease progression due to drug resistance. The underlying mechanisms of drug resistance in BC are still being explored, while those identified in prostate cancer may also apply to BC. In prostate cancer, the failure of AR-targeted therapy is related to AR gene amplification and mutation, interaction among coactivators, and overexpression of active AR splice variants, such as AR-V7 lacking the C-terminal LBD (91–96). Under the treatment of AR antagonists, overexpression, and structural mutation of AR make the receptor more sensitive to low levels of androgen and convert antagonist responses to agonistic ones (97–100). Different AR inhibitors may follow different resistance mechanisms. Recent studies found that the mutations of W741L and T877A mediate resistance to bicalutamide and hydroxyflutamide, respectively. A missense mutation of F876L in the LBD of AR was associated with the resistance to enzalutamide (76, 99–101).

Analysis of various splice variants revealed that 53.7% of primary BC samples had AR variant 7 (AR-V7) mutations (102). Interestingly, AR-V7, as AR splice variant in prostate cancer, is the most extensively researched among tumors and circulating tumor DNAs (103); it is widely accepted that monitoring and inhibiting AR-V7 is a clinical priority in prostate cancer treatment (104). Kohli et al. found that the AR variant 9 (AR-V9) is regularly co-expressed with AR-V7. Furthermore, high pre-therapy expression of AR-V9 mRNA in prostate cancer metastases was associated with the resistance to abiraterone acetate, indicating that AR-V9 might be a potential therapeutic target for drug resistance (104).

Moreover, recent studies have shown that the aberrant activation of the EMT and Wnt/β-catenin pathway also contributes to enzalutamide resistance in prostate cancer (105–107).

## Conclusions and Future Directions

AR is a burgeoning and promising therapeutic target that potentially improves the survival outcome of BC patients. This review emphasizes the role of AR and AR-related mechanisms in various BC subtypes from bench to bedside. However, additional researches are needed to elucidate the significance of AR, in particular, identification of appropriate AR detection methods and novel markers of AR responsiveness to better select the patients who may benefit from AR-related treatments. Up till now, there is no clinical guideline concerning the methods to test AR positivity. Immunohistochemistry (IHC) is the cheapest method and is widely used in the definition of AR positivity in BC (12). The optimal cut-off point has not yet been identified; the discrepancy rate of AR expression between primary BC and metastatic tissue, and the influence of multiple antibodies on AR status, make it hard to explain the role of AR or select patients for AR-targeted therapy according to the expression data obtained by IHC (108, 109). The characteristics of circulating tumor cells (CTCs) in blood, which are easily accessible through a simple venipuncture, may be a valuable alternative method to identify the AR status, especially in a metastatic tumor (110, 111). Hence, the differential expression of AR in patients with BC can be assessed at any time during follow-up and treatment (112). Of note, BC CTCs can also be used to evaluate the expression of AR-V7, as in prostate cancer, which may be a potential indicator for predicting the efficacy of abiraterone and enzalutamide (12, 103). In addition to expressing the AR protein, AR gene expression signatures, and AR phosphorylation status may be a predictor to define eligible patients for AR-targeted therapies (67, 113). Given the evidences reviewed herein, the combination of AR-targeted therapies with other therapies may improve the efficacy of BC treatment and hence, need to be explored further for their clinical effectiveness.

## Author Contributions

FQ and JH: conception and design. MC, YY, and KX: write, review, and revision of the manuscript. LL: supervision. All authors contributed to the article and approved the submitted version.

## Conflict of Interest

The authors declare that the research was conducted in the absence of any commercial or financial relationships that could be construed as a potential conflict of interest.

## Abbreviations

BC: breast cancer
ER: estrogen receptor
PR: progesterone receptor
HER2: human epidermal growth factor receptor 2
AR: androgen receptor
AR+: AR-positive
HER2+: HER2-positive
TNBC: triple-negative breast cancer
NTD: N-terminal domain
DBD: DNA-binding domain
LBD: ligand-binding domain
ARE: androgen response element
HSPs: heat shock proteins
ERE: estrogen response element
AKT: serine/threonine-protein kinase
FOXA1: forkhead box protein A1
mTOR: mammalian target of rapamycin
PTEN: phosphatase and tensin homolog
PI3K: phosphoinositide 3-kinase
GPER: G-protein coupled estrogen receptor
GPCR: G-protein coupled receptor
ARNILA: AR negatively-induced lncRNA
lncRNA: long non-coding RNA
EMT: epithelial–mesenchymal transition
DFS: disease-free survival
MBC: metastatic breast cancer
CBR: clinical benefit rate
PFS: progression-free survival
OS: overall survival
LAR: luminal androgen receptor
MSL: mesenchymal stem– like
BL2: basal-like 2
RB: retinoblastoma
PCR: pathologic complete response
CR or PR: complete response or partial response
RCB-I: residual cancer burden-index
CTCs: circulating tumor cells.
